# Left Ventricular Assist Device Implantation in Patients with Previous Open-Heart Surgery: Comparison of Median Sternotomy and Lateral Thoracotomy

**DOI:** 10.21470/1678-9741-2024-0210

**Published:** 2025-10-27

**Authors:** Cengiz Sahutoglu, Pelin Ozturk, Seden Kocabas, Fatma Zekiye Askar, Cagatay Engin, Tahir Yagdi, Mustafa Ozbaran

**Affiliations:** 1 Department of Anesthesiology and Reanimation, Ege University Medical Faculty, Izmir, Turkey; 2 Department of Cardiovascular Surgery, Ege University Medical Faculty, Izmir, Turkey

**Keywords:** Heart Failure, Heart-Assist Devices, Sternotomy, Minimally Invasive Surgical Procedures, Anesthetics, Analgesics.

## Abstract

**Introduction:**

Reoperative cardiac surgery is associated with a higher risk of complications due to technical difficulties compared to the first-time surgery. This study aims to compare the early outcomes of median sternotomy (MS) and lateral thoracotomy (LT) procedures in patients with a history of previous open-heart surgery who underwent left ventricular assist device (LVAD) implantation with cardiopulmonary bypass (CPB).

**Methods:**

A retrospective analysis was conducted on 36 patients who received LVAD implants for end-stage heart failure between November 2012 and June 2015. The patients were divided into Group 1 (MS, n = 18) and Group 2 (LT, n = 18).

**Results:**

The mean age of the patients was 57.2 ± 9.4 years (range: 24 - 70 years), and only 8.3% were female. Demographic data, preoperative characteristics, use of blood products, anesthetic drugs, and complications were similar in both groups (P > 0.05). The MS group had significantly longer operation duration (101 ± 46 minutes vs. 70 ± 20 minutes, P = 0.038) and CPB time (328 ± 79 minutes vs. 265 ± 47 minutes, P = 0.048) compared to the LT group. Postoperative analgesic consumption and pain scores were similar between the two groups (P > 0.05).

**Conclusion:**

In patients with a history of previous cardiac surgery, LVAD implantation with LT through CPB demonstrated favorable outcomes regarding reduced operation duration and CPB time. However, it did not positively impact the duration of stay in the intensive care unit, hospital stay, use of blood products, and complications.

## INTRODUCTION

**Table:** 

Abbreviations, Acronyms & Symbols				
BMI	= Body mass index		MIS	= Minimally invasive surgery
BSA	= Body surface area		MODS	= Multiple organ dysfunction syndrome
CPB	= Cardiopulmonary bypass		MS	= Median sternotomy
ECMO	= Extracorporeal membrane oxygenation		MV	= Mechanical ventilation
ES	= Erythrocyte suspension		NO	= Nitric oxide
EuroSCORE	= European System for Cardiac Operative Risk Evaluation		NSAIDs	= Non-steroidal anti-inflammatory drugs
FFP	= Fresh frozen plasma		PCWP	= Pulmonary capillary wedge pressure
ICU	= Intensive care unit		RVAD	= Right ventricular assist device
Intermacs	= Interagency Registry for Mechanically Assisted Circulatory Support		RVEF sPAP	= Right ventricular ejection fraction = Systolic pulmonary artery pressure
LT	= Lateral thoracotomy		TEE	= Transesophageal echocardiography
LVAD	= Left ventricular assist device		VAS	= Visual analog scale
LVEF	= Left ventricular ejection fraction			

Patients requiring re-sternotomy (redo surgery) during cardiac procedures are classified as high-risk due to associated technical challenges^[1^,^2]^. These difficulties, including limited access to the heart, prolonged surgery duration, and increased morbidity-mortality rates, are well-documented in this patient population. In addition, surgical and anesthesia teams must address concerns related to low cardiac function, massive blood transfusions, emergency cardiopulmonary bypass (CPB), total circulatory arrest, femoral-femoral bypass-related flow and perfusion failure, prolonged intensive care and hospital stays, and difficulties in accessing intravenous vasculature due to previous operations^[1^-^3]^.

In recent years, various minimally invasive techniques have been developed for left ventricular assist device (LVAD) implantation, which have gained popularity^[4]^. These methods can be performed with or without CPB. Bleeding and right heart failure after LVAD are important causes of mortality. Because the lateral thoracotomy (LT) approach involves less adhesion and less surgical trauma, the dissection is expected to be less bleeding. Additionally, right heart failure is less common with LVAD implantation due to reduced heart manipulation and shorter CPB time^[5]^. A comprehensive meta-analysis found that LVAD implantation via the LT reduced the rates of postoperative right ventricular failure and right ventricular assist device implantation, intensive care unit (ICU) stay, transfusion requirements, development of infection, and renal failure^[6^,^7]^. In some studies, it is stated that when median sternotomy (MS) and LT are compared, the results are similar after both LVAD and heart transplantation^[8^,^9]^.

While many studies have compared the MS and LT approaches to LVAD implantation, no study has focused on comparing the MS and LT approaches in patients with a history of previous open-heart surgery. In this study, we aim to compare the perioperative outcomes of the MS and the LT for LVAD implantation procedures in patients with a history of prior open-heart surgery. The study hypothesized that both groups would have similar complications depending on their previous surgeries.

## METHODS

### Study Design and Patient Selection

This retrospective, observational, cohort study included 36 patients who had a history of open-heart surgery and underwent LVAD implantation and CPB due to end-stage heart failure. The inclusion period for the patients was between November 2015 and March 2018. The study excluded patients without a history of cardiac surgery, those who underwent off-pump surgery, LVAD exchange, those implanted with an LVAD device other than HeartWare, and children. The patients were divided into two groups based on the surgical approach used for LVAD implantation: Group 1 (MS) consisted of 18 patients who underwent LVAD implantation with conventional MS, and Group 2 (LT) consisted of 18 patients who underwent LVAD implantation with the left anterolateral thoracotomy. The researchers obtained ethical approval from the Ege University Faculty of Medicine Clinical Research Committee (number: 18-4/44, date: 03/04/2018) before conducting the research. The study was conducted in line with the Helsinki Declaration principles.

### Anesthesia and Surgery Management

The same monitoring, anesthesia induction, anesthesia maintenance, anticoagulation treatment, and intensive care follow-up protocols were applied to all patients. All surgeries were performed by the same surgical team. During the surgical procedures, transesophageal echocardiography (TEE) was used to evaluate the heart. There may be some necessary concomitant procedures that need to be performed at the time of LVAD implantation, such as valve repair/replacement, or coronary bypass surgery. The surgical team preferred MS if concomitant procedures were required. In Group 1, MS and ascending aorta-right atrial cannulation were performed. In Group 2, a left anterolateral thoracotomy and left femoral artery-femoral vein cannulation were performed with the patient in the right lateral decubitus position. The left ventricular apex was incised, and the inflow of the LVAD was implanted. In Group 1, the outflow graft of the LVAD was anastomosed by placing a side clamp on the ascending aorta, while in Group 2, it was anastomosed on the descending aorta. The LVAD device was switched on after the cables were inserted. The location of the cannula and the volume status of the patients were evaluated using TEE, and inotropic/vasoconstrictor agents were adjusted to maintain a mean arterial pressure between 65 and 80 mmHg. All patients included in the study were transferred to the ICU with inotropic and vasoconstrictor support, nitroglycerin, and nitric oxide, which continued into the postoperative period.

### Data Collection

The researchers collected various data from the patients, including demographic characteristics, intraoperative hemodynamic and respiratory parameters, anesthetic and vasoactive drugs used, anesthesia and surgery times, use of blood products in the first 48 hours after surgery, mechanical ventilation time, length of hospital/ICU stay, postoperative complications, analgesics used and their amounts, and pain scores. Pain levels during the first 48 hours postoperatively were assessed using a visual analog scale (0 representing “no pain” and 10 being the “worst pain imaginable”) and a verbal rating scale (no pain, mild pain, moderate pain, severe pain), and the patient's highest pain score was recorded. Pain and analgesic use were obtained from the pain assessment form in the hospital's electronic management system. The study investigated several postoperative complications, including cardiac, respiratory, neurological, renal, gastrointestinal, hematologic, sepsis, multiple organ failure syndrome, and bleeding-related revision (HeartWare Inc., Framingham, MA, USA).

### Study Endpoints

The primary endpoint of the study was to compare the complications between the two groups, while the secondary endpoint was to assess postoperative pain levels and intraoperative parameters.

### Statistical Analysis

All statistical analyses were performed using IBM Corp. released 2012, IBM SPSS Statistics for Windows, version 21.0, Armonk, NY: IBM Corp. software. Continuous data were presented as mean ± standard deviation or median (minimum-maximum), while categorical variables were expressed as percentages (%). The normality of the data was evaluated using the Shapiro-Wilk test. Continuous variables were analyzed using the Student’s t-test or Mann-Whitney U test, and categorical variables were analyzed using the χ^^2^^ test or Fisher's exact test. A *P*-value < 0.05 was considered statistically significant.

## RESULTS

**Fig. 1:**
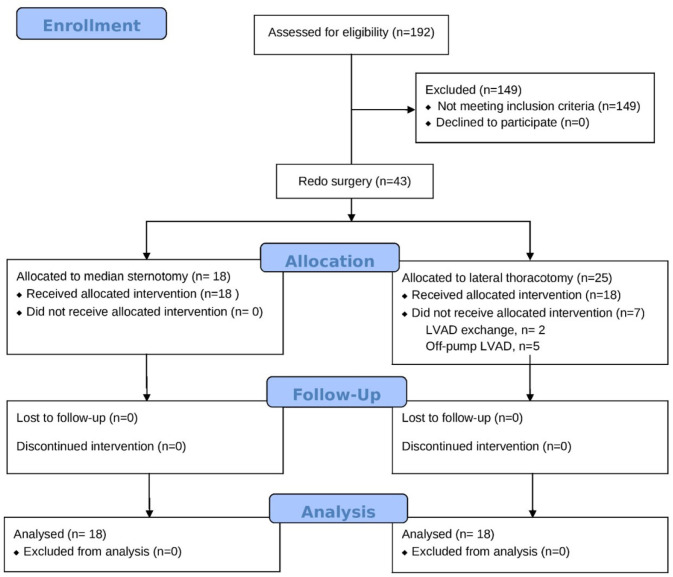
CONSORT flow diagram of the participants through each stage of the study. LVAD=left ventricular assist device.

The study examined the outcomes of patients who underwent LVAD surgery following previous open-heart procedures. A total of 36 patients met the study inclusion criteria out of the 190 patients who received the HeartWare LVAD (Figure 1). The mean age of the patients was 57.2 ± 9.4 years, and only 8.3% were female. The demographic and preoperative characteristics of the patients were comparable in both groups and are presented in Table 1 (*P* > 0.05).

**Table 1:** Preoperative data and demographic characteristics of the patients.

	Sternotomy	Thoracotomy	*P*-value
	(n = 18)	(n = 18)	
Age (years)	55.1 ± 11.1	59.3 ± 7.1	0.182
Sex (male) (%)	17 (94.4)	16 (88.9)	0.546
BMI (kg/m^2^)	24.5 ± 2.9	26.3 ± 5.1	0.184
BSA (m^2^)	1.84 ± 0.17	1.9 ± 0.23	0.495
LVEF (%)	21 ± 4	21 ± 5	0.907
RVEF (%)	42 ± 6	37 ± 6	0.056
sPAP (mmHg)	49 ± 21	41 ± 12	0.213
PCWP (mmHg)	28.6 ± 7.9	28.6 ± 5.8	0.988
EuroSCORE	12 ± 4.3	13.8 ± 2.5	0.237
Intermacs	3 (1 - 4)	3.5 (2 - 4)	0.188
Reason for LVAD implantation (n, %)			
Ischemic cardiomyopathy	9 (64.3)	5 (35.7)	0.171
Dilated cardiomyopathy	9 (40.9)	13 (59.1)	
Previous cardiac surgery (n, %)			
Coronary artery bypass grafting	12 (54.5)	10 (45.5)	
Heart valve surgery	4 (57.1)	3 (42.9)	0.447
Other^*^	2 (28.6)	5 (71.4)	

The data were presented as either mean ± standard deviation, median (minimum-maximum), or number of patients (percent).

* Multiple cardiac surgeries, aortic surgery

BMI=body mass index; BSA=body surface area; EuroSCORE=European System for Cardiac Operative Risk Evaluation; Intermacs=Interagency Registry for Mechanically Assisted Circulatory Support; LVAD=left ventricular assist device; LVEF=left ventricular ejection fraction; PCWP=pulmonary capillary wedge pressure; RVEF=right ventricular ejection fraction; sPAP=systolic pulmonary artery pressure

The administration of fentanyl (403 ± 87 µg *vs.* 360 ± 135 µg, *P* = 0.250), rocuronium (170 ± 21 mg *vs.* 166 ± 37 mg, *P* = 0.683), ketamine (100 mg [50 - 200] *vs.* 100 mg [80 - 150], *P* = 0.988), and midazolam (3 mg [2 - 5] *vs.* 4 mg [2 - 10], *P* = 0.317) was comparable in both groups. During the surgery, three patients experienced difficulty with surgical cannulation (2 *vs.* 1, *P* > 0.99), four patients had desaturation (0 *vs.* 4, *P* = 0.104), three patients had intraoperative hypotension (2 *vs.* 1, *P* > 0.99), and one patient suffered cardiac arrest (1 *vs.* 0, *P* > 0.99) in the MS and LT groups, respectively. The duration of the operation and CPB time were found to be shorter in the LT group (Group 2) compared to the MS group (Group 1). Intraoperative and postoperative use of blood products and the amount of bleeding were similar in both groups (Table 2).

**Table 2:** Intraoperative and postoperative parameters of the patients.

	Sternotomy	Thoracotomy	*P*-value
	(n = 18)	(n = 18)	
Cardiopulmonary bypass (min.)	101 ± 46	70 ± 20	0.048
Duration of operation (min.)	328 ± 79	265 ± 47	0.038
Duration of anesthesia (min.)	361 ± 76	316 ± 48	0.107
Mechanical ventilation (hour)	26 (8 - 190)	21.5 (12 - 251)	0.438
Duration of the ICU stay (day)	5 (3 - 20)	5 (3 - 21)	0.443
Duration of hospital stay (days)	22.1 ± 11	21.1 ± 10.6	0.771
Intraoperative whole blood (U)	0.7 ± 1.1	1 ± 0.9	0.317
Intraoperative ES (U)	0 (0 - 7)	0 (0 - 1)	0.851
Intraoperative FFP (U)	0 (0 - 4)	1 (0 - 2)	0.443
Intraoperative thrombocyte (U)	2 ± 3.6	1.5 ± 2.7	0.631
Postoperative 24-hour whole blood (U)	1.4 ± 0.9	1.3 ± 1.3	0.893
Postoperative 24-hour ES (U)	0.5 (0 - 2)	0.5 (0 - 5)	0.976
Postoperative 24-hour FFP (U)	2.1 ± 1.1	1.8 ± 1.4	0.566
Postoperative 24-hour thrombocyte (U)	3 (0 - 6)	0 (0 - 6)	0.350
Drainage amount (first 24 hours) (mL)	693 ± 338	592 ± 387	0.571

The data were expressed as either mean ± standard deviation or median (minimum-maximum)

ES=erythrocyte suspension; FFP=fresh frozen plasma; ICU=intensive care unit

There were no significant differences between the two groups in terms of the use of inotropic or vasoconstrictor agents, nitroglycerin, and nitric oxide (*P* > 0.05). Overall, 69.4% of patients developed at least one postoperative complication, but there was no significant difference observed between the groups in terms of complication development (66.7% *vs.* 72.2%, *P* = 0.717). The primary cause of death was right heart failure in eight patients (5 *vs.* 3, *P* = 0.747), and multiple organ dysfunction syndrome (MODS) or sepsis in two patients (1 *vs.* 1, *P* > 0.99). The discharge rate following redo LVAD surgery was 72.2% (66.7% *vs.* 77.8%, *P* = 0.457) (Table 3).

**Table 3:** Patient groups and complications.

Complications (n, %)	Sternotomy	Thoracotomy	*P*-value
	(n = 18)	(n = 18)	
At least one complication	12 (66.6)	13 (72.2)	0.717
Cardiac	8 (44.4)	6 (33.3)	0.494
Right ventricular failure	6 (33.3)	3 (16.7)	0.443
RVAD/ECMO	3 (16.7)	0 (0)	0.229
Use of NO	5 (27.8)	7 (38.9)	0.480
Inotropes for > 14 days	4 (22.2)	3 (16.7)	0.674
Respiratory	8 (44.4)	6 (33.3)	0.494
> 2 days MV	6 (33.3)	5 (27.8)	0.717
Pneumonia	6 (33.3)	5 (27.8)	0.717
Neurological	0 (0)	4 (22.2)	0.104
Ischemic stroke	0 (0)	3 (16.7)	0.229
Renal	5 (27.8)	8 (44.4)	0.298
Hemodialysis	5 (27.8)	5 (27.8)	> 0.99
Gastrointestinal	4 (22.2)	3 (16.7)	0.674
Hematologic	2 (11.1)	2 (11.1)	> 0.99
MODS	5 (27.8)	4 (22.2)	0.700
Sepsis	5 (27.8)	3 (16.7)	0.691
Bleeding-related revision	2 (11.1)	2 (11.1)	> 0.99
Mortality	6 (33.3)	4 (25)	0.457

Multiple complications were observed in most of the patients. The data were expressed as the number of patients and the corresponding percentage

ECMO=extracorporeal membrane oxygenation; MODS=multiple organ dysfunction syndrome; MV=mechanical ventilation; NO=nitric oxide; RVAD=right ventricular assist device

Regarding postoperative pain, 72.2% of the total patients experienced either no pain or only mild pain (visual analog scale < 4) within the first 48 hours. Seven patients did not require any postoperative analgesics. Among the remaining 29 patients, nine were treated with tramadol alone, four with paracetamol alone, 14 with tramadol + paracetamol, and two with tramadol + paracetamol + non-steroidal anti-inflammatory drugs (NSAIDs). Postoperative analgesic consumption and pain scores were similar in both groups (Table 4).

**Table 4:** Analgesic requirements and pain levels of the patients within the first 48 hours after the operation.

	Sternotomy	Thoracotomy	*P*-value
	(n = 18)	(n = 18)	
Postoperative			
Tramadol (mg)	156 ± 138	150 ± 209	0.926
Paracetamol (gr)	1 (0 - 4)	0 (0 - 7)	0.323
Visual analog scale (maximum)	2 (0 - 8)	1 (0 - 10)	0.203
Verbal rating scale			
None (VAS: 0)	3 (16.7)	4 (22.2)	
Mild (VAS: 1 - 3)	9 (50.0)	10 (55.6)	0.831
Moderate (VAS: 4 - 6)	4 (22.2)	2 (11.1)	
Severe (VAS: 7 - 10)	2 (11.1)	2 (11.1)	

The data were presented using various statistical measures including mean ± standard deviation, median (minimum-maximum), or number of patients (percent)

VAS=visual analog scale

## DISCUSSION

This study aimed to evaluate the outcomes of LVAD implantation in patients with a history of cardiac surgery. The utilization of LT with CPB was found to reduce the duration of surgery and bypass time. Nevertheless, this approach did not significantly affect the occurrence of complications or pain scores.

Repeat cardiac surgery, also known as redo surgery, presents technical challenges due to potential issues such as tissue scarring, loss of tissue layers, adhesion, and the risk of injury to bypassed coronaries and large vessels during reentry. Minimally invasive approaches are considered preferable for redo surgery as they involve a lower risk of dissection and trauma. Previous studies on cardiac valve surgeries have reported an increase in mortality rates of 11.1% and 13.4%, and a 2.6-fold increase in mortality risk associated with re-sternotomy. Therefore, it is crucial to establish safe strategies in the preoperative period to reduce injury rates in these patients^[10^-^12]^.

In our study, we employed anesthetic agents and nitroglycerin to reduce blood pressure by lowering systolic arterial pressure to 80 - 90 mmHg. Despite these efforts, emergency CPB was required for two patients who underwent a sternotomy. Among three patients who underwent a left anterior thoracotomy, lung injuries occurred. One patient underwent wedge resection, while the other two patients had their lungs repaired with primary sutures. Patients who experienced hypoxemia had their mechanical ventilation settings readjusted. Initially, positive end-expiratory pressure values (up to 8 cm H₂O) and FiO₂ values (up to 100% if necessary) were increased, and the endotracheal tube was checked for patency through in-tube aspiration. In non-responsive patients, normal tidal volume was resumed after consultation with the surgical team. Before closing the thorax/mediastinum and leaving the operating room, manual recruitment maneuvers (10 - 15 sec and 35 cm H₂O) were performed in all patients to prevent atelectasis based on their hemodynamics.

In a meta-analysis of 1,586 patients who underwent redo aortic valve surgery, Phan et al.^[13]^ compared minimally invasive surgery (MIS) and conventional surgery (MS) methods. Both groups demonstrated comparable outcomes in terms of CPB time and aortic cross-clamping time, ICU and hospital stay, operative mortality, bleeding-related revision, pacemaker requirement, acute renal failure, and stroke development. Dieberg et al.^[14]^ reported that MIS resulted in shorter durations of CPB, aortic cross-clamping, and operation, as well as reduced length of hospitalization when early extubation was applied. However, postoperative complications were comparable between the two groups. The findings indicated that MIS reduced treatment costs due to a decrease in ICU stay duration. In contrast, Sharony et al.^[15]^ conducted a study involving 498 patients comparing minimally invasive redo-valve surgery (MIS) (n = 161) and the MS group (n = 337). The MIS group exhibited lower in-hospital mortality (3% *vs.* 14%), wound infection rates (0% *vs.* 2.4%), blood product use, and duration of hospital stay. CPB, aortic cross-clamping time, and stroke development were similar in both groups.

Rojas et al.^[16]^ conducted a study involving 46 patients, aged over 60 years old, comparing MIS and MS. The study found that the MIS group had lower rates of postoperative bleeding (0% *vs.* 26%, *P* = 0.014), which required revision, and postoperative prolonged inotropic support (5% *vs.* 34.6%, *P* = 0.028). Other complications were similar between the two groups (85% *vs.* 69.2%, *P* = 0.302). The discharge rates were reported as 85% for the MIS group and 76.9% for the MS group (*P* = 0.71). The primary causes of mortality were right heart failure (33%), sepsis (33%), and MODS (33%) in the MIS group, while they were intracranial hemorrhage (25%), sepsis (25%), MODS (25%), right heart failure (12.5%), and bleeding-related revision (12.5%) in the MS group. Vinogradsky et al.^[17]^ found no differences between the LT and the MS in ICU or hospital length of stay, extubation time, ICU readmission rate, vasoactive inotropic scores, or in-hospital mortality. The study reported that patients who underwent the LT had significantly less early right ventricular failure in the acute phase (24.4% *vs.* 53.7%, *P* = 0.004). However, this group had a higher incidence of tricuspid regurgitation in the long term. The authors concluded that although the LT for LVAD implantation is a safe alternative to the MS, it is not superior in terms of perioperative and postoperative outcomes. Gosev et al.^[18]^ found no significant differences between the two groups in blood product use, adverse events (including right heart failure), functional status, or quality of life.

In our study, all patients underwent CPB. The application of LVAD with LT reduced the duration of operation and the CPB time. However, it did not have a positive impact on blood usage, mechanical ventilation time, ICU stay, and hospital length of stay. Sahutoglu et al.^[9]^ reported that LVAD implantation with CPB increased the duration of operation time and postoperative blood product usage but did not affect complications. Previous pleural opening and thoracic tube applications resulted in thoracic adhesions in the patients, making surgical dissection challenging and causing lung injuries. Additional interventions performed on the lung may have prolonged the operation time, which could explain the lack of difference between the groups. Despite the extended CPB duration associated with the need for additional intervention, this method is currently considered the most reliable. In our institution, the in-hospital mortality rate following LVAD implantation was 17.9%, compared to 28.9% in those who underwent redo surgery.

Thoracic surgery often leads to severe pain, which can negatively impact respiratory functions and the patient's quality of life. Therefore, it is crucial to implement dynamic analgesia to enable post-thoracotomy patients to cough and breathe deeply. A variety of techniques are employed to control pain in thoracotomy patients, including epidural analgesia, paravertebral or intercostal blocks, intrathecal or epidural morphine administration, and systemic analgesics (such as NSAIDs, paracetamol, and opioids)^[19]^. Ahangar et al.^[20]^ demonstrated that aortic valve replacement performed with a right anterolateral thoracotomy is less painful than the MS (mean pain score 4.2 ± 0.6 *vs.* 5.4 ± 0.6, *P* < 0.001). In a study by Walther et al.^[21]^, the mean postoperative pain scores for the MIS and the MS were found to range from 4 to 6.5. Patients who underwent left anterolateral thoracotomy experienced the most acute pain during the first two days. After the third postoperative day, pain levels were lower in the MIS bypass and MIS mitral valve surgery groups compared to the conventional group, but higher in the MIS aortic valve surgery group. No reduction in pain levels was observed after the fifth postoperative day in the MIS bypass, MIS mitral valve, or MIS aortic valve operation groups compared to the conventional group.

In our patient population, we observed no increase in pain scores or analgesic consumption because of the thoracotomy procedure. Administration of ketamine and fentanyl during anesthesia induction resulted in reduced early analgesic requirements. Postoperative pain management was achieved effectively using second-line analgesics. The incorporation of analgesic drugs such as lidocaine and magnesium, which also possess antiarrhythmic properties, contributed to low pain scores. However, it should be noted that thoracic epidural and regional blocks (such as paravertebral or intercostal blocks), known to be the optimal analgesic methods for thoracic surgery, were not employed in 65.8% of our patients due to concerns regarding the potential bleeding risk associated with anticoagulants and antiaggregants.

### Limitations

This study has several limitations. Firstly, it was a retrospective study encompassing only a small patient cohort. Therefore, we wanted to present our current experience. It needs to be supported by large series of randomized controlled trials. Secondly, patients who required additional intervention (coronary bypass or valve replacement) were included in the MS group. Therefore, the operation time may have been longer in the MS group. Third, the patients had a short duration of follow-up. The impact of these methods on heart transplantation and life expectancy should also be studied. Lastly, while the investigation focused on acute pain, chronic pain was not evaluated.

## CONCLUSION

MS remains the most prevalent approach for patients requiring additional cardiac intervention in LVAD implantation. Although the combined LT and LVAD implantation with CPB may reduce the duration of operation and CPB time, it did not improve the duration of stay in the ICU, hospital stay, use of blood products, and complications. In addition, the postoperative pain scores and the analgesic consumption in the LT group were similar to the MS group. However, further investigation through large randomized controlled trials is needed to confirm our results.

## Data Availability

The authors declare that the data will be available upon reasonable request to the corresponding author.

## Artificial Intelligence Usage

The authors declare use of ChatGPT (OpenAI) for English language editing. The content produced by the artificial intelligence tool was revised and edited by the authors as necessary, and they take full responsibility for the content to be published.

## References

[1] Luciani N, Nasso G, Anselmi A, Glieca F, Gaudino M, Girola F (2006). Repeat valvular operations: bench optimization of conventional
surgery. Ann Thorac Surg.

[2] Jones JM, O'kane H, Gladstone DJ, Sarsam MA, Campalani G, MacGowan SW (2001). Repeat heart valve surgery: risk factors for operative
mortality. J Thorac Cardiovasc Surg.

[3] Rupprecht L, Schopka S, Keyser A, Lunz D, Sossalla S, Hilker M (2022). 25 years' experience with redo operations in cardiac
surgery-third-time sternotomy procedures. Thorac Cardiovasc Surg.

[4] Maltais S, Davis ME, Haglund N. (2014). Minimally invasive and alternative approaches for long-term LVAD
placement: the Vanderbilt strategy. Ann Cardiothorac Surg.

[5] Cheung A, Lamarche Y, Kaan A, Munt B, Doyle A, Bashir J (2011). Off-pump implantation of the HeartWare HVAD left ventricular
assist device through minimally invasive incisions. Ann Thorac Surg.

[6] Mariani S, Li T, Boethig D, Napp LC, Chatterjee A, Homann K (2021). Lateral thoracotomy for ventricular assist device implantation: a
meta-analysis of literature. ASAIO J.

[7] Zubair MH, Brovman EY. (2023). Lateral thoracotomy versus sternotomy for left ventricular assist
device implantation. Curr Opin Anaesthesiol.

[8] Hironaka CE, Deng B, Kawabori M, Critsinelis AC, Zhan Y, Chen FY (2021). Left thoracotomy vs full sternotomy for centrifugal durable LVAD
implantation: 1-year outcome comparison post-LVAD and post-heart
transplantation. J Artif Organs.

[9] Sahutoglu C, Turksal E, Bilic U, Kocabas S, Zekiye Askar F, Ozturk P (2017). Anesthetic management for left ventricular assist device
implantation through left thoracotomy: evaluation of on-pump versus
off-pump. Transplant Proc.

[10] Imran Hamid U, Digney R, Soo L, Leung S, Graham AN. (2015). Incidence and outcome of re-entry injury in redo cardiac surgery:
benefits of preoperative planning. Eur J Cardiothorac Surg.

[11] Toker ME, Eren E, Guler M, Kirali K, Yanartas M, Balkanay M (2009). Second and third cardiac valve reoperations: factors influencing
death and long-term survival. Tex Heart Inst J.

[12] Balsam LB, Grossi EA, Greenhouse DG, Ursomanno P, Deanda A, Ribakove GH (2010). Reoperative valve surgery in the elderly: predictors of risk and
long-term survival. Ann Thorac Surg.

[13] Phan K, Zhou JJ, Niranjan N, Di Eusanio M, Yan TD. (2015). Minimally invasive reoperative aortic valve replacement: a
systematic review and meta-analysis. Ann Cardiothorac Surg.

[14] Dieberg G, Smart NA, King N. (2016). Minimally invasive cardiac surgery: a systematic review and
meta-analysis. Int J Cardiol.

[15] Sharony R, Grossi EA, Saunders PC, Schwartz CF, Ursomanno P, Ribakove GH (2006). Minimally invasive reoperative isolated valve surgery: early and
mid-term results. J Card Surg.

[16] Rojas SV, Hanke JS, Avsar M, Ahrens PR, Deutschmann O, Tümler KA (2018). Left ventricular assist device therapy for destination therapy:
is less invasive surgery a safe alternative?. Rev Esp Cardiol (Engl Ed).

[17] Vinogradsky A, Ning Y, Kurlansky P, Kirschner M, Yuzefpolskaya M, Colombo P (2024). Less is better? Comparing effects of median sternotomy and
thoracotomy surgical approaches for left ventricular assist device
implantation on postoperative outcomes and valvulopathy. J Thorac Cardiovasc Surg.

[18] Gosev I, Pham DT, Um JY, Anyanwu AC, Itoh A, Kotkar K (2024). Ventricular assist device using a thoracotomy-based implant
technique: multi-center Implantation of the HeartMate 3 in subjects with
heart failure using surgical techniques other than full median sternotomy
(HM3 SWIFT). J Thorac Cardiovasc Surg.

[19] Kolettas A, Lazaridis G, Baka S, Mpoukovinas I, Karavasilis V, Kioumis I (2015). Postoperative pain management. J Thorac Dis.

[20] Ahangar AG, Charag AH, Wani ML, Ganie FA, Singh S, Ahmad Qadri SA (2013). Comparing aortic valve replacement through right anterolateral
thoracotomy with median sternotomy. Int Cardiovasc Res J.

[21] Walther T, Falk V, Metz S, Diegeler A, Battellini R, Autschbach R (1999). Pain and quality of life after minimally invasive versus
conventional cardiac surgery. Ann Thorac Surg.

